# The Treatment of Diphtheria

**Published:** 1906-08-04

**Authors:** J. W. Miller

**Affiliations:** Assistant Medical Officer of Health, Blackburn.


### THE TREATMENT OF DIPHTHERIA.

By J. W. Miller, M.D.(Vict.), D.P.H.(Camb.), Assistant Medical Officer of Health, Blackburn.

Statistics have shown that in cases in which antitoxin is injected early, there is less chance of paralysis afterwards following, and should it occur the paralysis is not so marked as in cases in which the antitoxin has either been injected late or not at all.

With regard to the dosage of antitoxin, in mild cases seen on the first or second day, one injection of 4,000 units may be sufficient. In cases of moderate severity, seen on the first or second day, a dose of 6,000 to 8,000 units is often sufficient. In severe cases, and in all cases treated on the third day or later, 8,000 units should be given immediately, and this dose repeated at intervals of 12 hours if the membrane is not separating and signs of improvement are not apparent. In some cases a third injection may be necessary, and rarely a fourth.

Where there is croup 8,000 units should be injected as soon as the case is seen, followed by another 8,000 units in the majority of cases, and a third injection in severe cases. In all doubtful cases, unless there is very little affection of the throat, and in all cases of suspicious croup, 4,000 units should be injected before waiting for the result of the bacteriological examination. Even 2,000 units injected may be of the greatest service, especially in cases of croup where the disease advances rapidly, and this amount can be injected by two fillings of an ordinary hypodermic syringe.

The size of the dose should depend on the severity of the case, and not on the age of the patient; in fact, children often need a larger dose than adults, as there is greater constitutional disturbance than in an adult. In addition to being used as a curative agent, diphtheritic antitoxin can also be used as a prophylactic remedy in persons exposed to infection, an injection of 500 units being sufficient to protect for three weeks.

Antitoxin should always be injected and never given by the mouth, either side of the abdomen being the most suitable site.

It is important that care should be taken in regard to the sterilisation of the needle and syringe, as organisms will readily grow in the serum. There are no contra-indications to the injection of antitoxin, and a large dose can be injected in an infant without ill effect. In about 50 to 60 per cent. of cases injected with antitoxin a rash appears from the seventh to the twelfth day, occasionally as early as the second or third, or as late as the third week after injection. The rash usually lasts several days, and sometimes fades away and reappears again; it is generally accompanied by a rise of temperature, but there is little if any constitutional disturbance.

The rash may be limited to the site of injection; it then usually appears about the second or third day, but in the majority of cases it is present over the trunk and extremities and sometimes the face, often starting at the site of injection and extending to other parts of the body.

The most common varieties of rash are erythema multiforme, and urticaria; sometimes the rash resembles that of measles or scarlet fever, more often the former. The diagnosis from these two diseases is usually easy; the antitoxin rash is evanescent, and generally first seen at the site of injection. Often two varieties of rash are seen in the same patient; ii the rash is erythematous an urticarial patch can usually be found somewhere. Other symptoms associated with measles or scarlet fever are absent. When desquamation occurs it resembles that following measles.

Joint pains, or more commonly pains in the tissues around the joints, usually the knee, occur in about 6 per cent. of cases injected in association with the rash. The pain is more effectually relieved by local applications than the internal administration of salicylate of soda.

The antitoxin rash is probably the result of the horse's serum alone, as injection of ordinary horse serum will produce the rash. Since the antitoxin has been preferred in a more concentrated form, a less amount of serum has been injected, and the rash has occurred less frequently and has been less intense.

After antitoxin the next most important treatment is rest. In all cases of diphtheria, including the mildest, the patient should be kept in the recumbent position for at least three weeks, and then only allowed to sit up if no irregularity of the pulse be present. At first the patient should be supported by pillows in a half-recumbent position for about 20 minutes on the first day, and on the second or third day should be allowed to sit up, at first for about half an hour, this period being increased, and the patient allowed up for a short time at the end of a few days, the length of time being gradually increased. Muscular exertion should be avoided, and should vomiting or faintness come on, indicating cardiac weakness, the patient should be kept in bed in the recumbent position for a longer period, and the same precautions used as before. In cases in which paralysis is confined to the palate or ciliary muscles, a full month should be allowed in the recumbent position, and then the patient should only be propped up if the pulse is regular. In cases of generalised paralysis a longer period is necessary, and on no account in any case of diphtheria, whether mild or severe, should a patient be allowed up so long as any irregularity of the pulse is present, even if this necessitates remaining in bed for eight or nine weeks or longer, as death has occurred as late as the tenth week from sudden strain on the heart. The diet in diphtheria should be stimulating, milk being supplemented by beef tea and the various peptonoids. Solid food should be given as soon as the condition of the throat will allow.

The presence of a small amount of albumen in the urine does not contra-indicate the use of eggs and farinaceous food; it is most important that nourishing food should be administered in order that the general strength may be maintained.

Where there is regurgitation on the swallowing of liquids, the milk should be thickened with Benger's food or arrowroot, and if this cannot be swallowed, blanc mange made of corn flour and milk, isinglass containing beef tea, and other preparations, or lightly boiled eggs made into a paste with bread crumbs should be tried, as solids can often be swallowed when the taking of liquids is impossible. In the worst cases of regurgitation, and in some laryngeal cases, nasal feeding will have to be resorted to, and if this is unsuccessful, as a last resource rectal feeding; this latter method of feeding is very unsatisfactory.

With regard to the occurrence of frequent vomiting usually accompanying other signs of heart failure, no drug appears to have any marked effect. A mustard plaster over the epigastrium sometimes has a beneficial effect. Stimulants should be given in all cases with weak or irregular action of the heart. In the early stages the best stimulant is brandy or whisky; ether and camphor are also useful. Any stimulants which either tend to raise the blood-pressure or act on the inhibiting centres of the heart—*e.g.* digitalis—are contra-indicated. Throughout the disease strychnine can be given internally or by hypodermic injection.

Small quantities of atropine are recommended by Bolton in cases of heart failure, on account of the action of this drug in paralysing the terminations of the vagi nerves in the heart, and so accelerating the beat and increasing the systole. Where paralysis is present a combination of strychnine and iron is useful.

Strychnine should be injected hypodermically on the occurrence of symptoms of heart failure. Rolleston recommends adrenalin chloride 1 in 1,000 in doses of five to ten minims, four hourly, increased in some cases to as much as one or two drachms, four hourly, in cases of heart failure. In the early stages of the disease there is a tendency, especially in children, to vomit after medicine; purgatives should therefore be given in a form easily taken—*e.g.* in capsuloids or tabloids; in some cases it is preferable to give an enema.

The early injection of antitoxin will often prevent the necessity of tracheotomy in laryngeal cases. The outer tube needs changing at least once, sometimes oftener, in the 24 hours by the medical attendant, and the inner tube several times by the nurse. Sometimes the tube is coughed out of the trachea, and under these circumstances, or if the tube got blocked up with membrane and mucus, suffocation would quickly take place if there were no one there to hold the trachea open with forceps or clean out the tracheotomy tube.

Tracheotomy should be performed as soon as possible when the conditions demand it, as cases do occasionally recover even under the worst conditions, although the mortality from tracheotomy cases is high. Several hours in a steam tent will often cause considerable improvement in the breathing.

*Local treatment.*—Antitoxin not only neutralises the toxins produced by the bacillus, but also has a local effect, causing the membrane to shrivel up and separate. If the membrane is not separating readily the antitoxin should be repeated.

Boracic lotion in the milder cases, and chlorine water in cases where there is much discharge from the throat, should be applied to the throat, four hourly for the first few days, either by gentle syringing or swabbing. If there be much struggling, and this often occurs with young infants, the throat should be disturbed as little as possible, being swabbed out occasionally, as the injection of antitoxin will cause the membrane to separate.

With extensive membrane which does not readily separate, in addition to repeated injections of antitoxin, a solution of 20 to 30 per cent. carbolic acid in glycerin or biniodide of mercury 1 in 1,000 should be applied to the throat. Local treatment of the throat should be continued for some days after the membrane has disappeared, to prevent its reappearance, and swabbed or painted daily up to the end of the third week. By this time all diphtheria bacilli will usually have disappeared from the throat. In only a small percentage of cases are the bacilli absent at the end of a fortnight. Where the bacilli persists in the throat, their disappearance can be hastened by the application of biniodide of mercury, 1 in 1,000, or izal 1 in 500, applied daily. Patients should be isolated until the disappearance of diphtheria bacilli from the throat, and also the nose or ears, should any discharge have been present.

It is important that swabs should be taken from the throats of persons in the same house as a diphtheria patient on the slightest suspicion of any affection, even when no membrane can be detected. Should diphtheria bacilli be found, such persons should be isolated until their disappearance from the throat. Infection is sometimes conveyed by healthy persons who have been in contact with cases of diphtheria but without any signs of the disease acting as carriers, the bacilli being present in their throats.

In regard to these persons, if isolation is difficult, the throat should be gargled or local antiseptics applied until a swab is returned as negative.

In taking a swab it is important that no antiseptic should have been applied to the throat for several hours previously, as recent application of an antiseptic may prevent the growth of the diphtheria bacilli. By means of early bacteriological diagnosis valuable assistance is afforded not only in the prevention of the disease, but also in rendering imperative the injection of antitoxin at the earliest possible moment.

